# Supplementary material for: Shared but Clean Household Toilets: What Makes This Possible? Evidence from Ghana and Kenya
Source: Int J Environ Res Public Health. 2022 Apr 2;19(7):4271. doi: 10.3390/ijerph19074271 (PMC8998870; doi:10.3390/ijerph19074271)
Supplement: Supplementary file 1 [file ijerph-19-04271-s001.zip › ijerph-1592979-supplementary.pdf]

International Journal of
Environmental Research and Public Health

MDPI

Shared but Clean Household Toilets: What Makes This Possible? Evidence from Ghana and Kenya

Supplementary Materials

Table S1. Indicators of quality of shared sanitation facility.

Quality indicators	Scale	Ghana		Kenya	
**Hygiene**		Yes [n (%)]	No [n (%)]	Yes [n (%)]	No [n (%)]
Is there faecal matter on the slab/seat?	Yes = 0 No = 1	49 *(12)*	362 *(88)*	76 *(17)*	362 *(83)*
Are there flies on the facility	Yes = 0 No = 1	101 *(25)*	310 *(75)*	407 *(93)*	31 *(7)*
Is there a noticeable odour on the facility?	Yes = 0 No = 1	137 *(33)*	274 *(67)*	410 *(94)*	28 *(6)*
Is there urine/saliva on the facility?	Yes = 0 No = 1	25 *(6)*	386 *(94)*	216 *(49)*	222 *(51)*
Are there maggots in the privy room of the facility?	Yes = 0 No = 1	29 *(7)*	382 *(93)*	28 *(6)*	410 *(94)*
Are there rodents on the facility?	Yes = 0 No = 1	25 *(6)*	386 *(94)*	22 *(5)*	416 *(95)*
Hygiene score [Median (95%CI)]		6 (4.99 – 5.23)		3 (2.54 – 2.73)	
**Privacy**					
Does the toilet facility have a door?	Yes = 1 No = 0	393 (96)	18 (4)	397 (91.7)	36 (8.3)
Does the door have a locking latch on the inside?	Yes = 1 No = 0	319 (78)	92 (22)	198 (45.7)	235 (54.3)
Does the door have a locking latch on the outside?	Yes = 1 No = 0	376 (91)	35 (9)	334 (77.1)	99 (22.9)
Does the door offer privacy?	Yes = 1 No = 0	343 (83)	68 (17)	376 (86.8)	57 (13.2)
Does the superstructure offer privacy?	Yes = 1 No = 0	340 (83)	70 (17)	329 (76.5)	101 (23.5)
Privacy score [Median (95%CI)]		5 (4.20 – 4.42)		4 (3.65 – 3.90)	
**Accessibility**					
Does everyone in the house use the toilet facility?	Yes = 1 No = 0	398 (97)	13 (3)	390 (90.7)	40 (9.3)
Is the toilet facility accessible at all times?	Yes = 1 No = 0	407 (99)	3 (1)	330 (76.6)	101 (23.4)
Accessibility score [Median (95%CI)]		2 (1.94 – 1.98)		2 (1.62 – 1.72)	

Table S2. Predictors of total quality of shared sanitation.

Predictors	β	OR	SE	P (95% CI)
Presence of functional out-side door lock	1.71	5.53	0.19	<0.05 (1.34–2.08)**
Type of sanitation facility	2.04	7.69	0.15	<0.05 (1.74–2.34)**
Cleaning toilet daily	0.28	1.32	0.15	0.06 (-0.02– 0.57)
Involving landlords in cleaning toilet facility	-0.11	0.90	0.23	0.61 (-0.56 – 0.33)
Presence of landlord	1.14	3.13	0.14	<0.05 (0.85–1.41)**
Number of households sharing toilet facility	0.001	1.00	0.01	0.94 (-0.03–0.03)
R-squared			0.36	

** statistically significant predictor of the overall quality of shared sanitation.
